# Evolution of genetic markers for drug resistance after the introduction of dihydroartemisinin–piperaquine as first-line anti-malarial treatment for uncomplicated falciparum malaria in Indonesia

**DOI:** 10.1186/s12936-023-04658-4

**Published:** 2023-08-09

**Authors:** Farindira Vesti Rahmasari, Puji Budi Setia Asih, Ismail Ekoprayitno Rozi, Suradi Wangsamuda, Rifqi Risandi, Farahana Kresno Dewayanti, Dendi Hadi Permana, Lepa Syahrani, Helen Dewi Prameswari, Herdiana H. Basri, Maria Dorina G. Bustos, Prakaykaew Charunwatthana, Arjen M. Dondorp, Mallika Imwong, Din Syafruddin

**Affiliations:** 1 Graduate Programme in Molecular Medicine, Faculty of Science, Mahidol University, Bangkok, 10400 Thailand; 2 Department of Molecular Tropical Medicine and Genetics, Faculty of Tropical Medicine, Mahidol University, 420/6 Ratchawithi Road, Ratchathewi, Bangkok, 10400 Thailand; 3 Department of Parasitology, School of Medicine, Faculty of Medicine and Health Sciences, Universitas Muhammadiyah Yogyakarta, Bantul, Indonesia; 4 Eijkman Research Center for Molecular Biology, National Research and Innovation Agency (BRIN), Cibinong, Indonesia; 5 Malaria Working Group, Ministry of Health, Jakarta, Indonesia; 6 World Health Organization, Country Office for Indonesia, Jakarta, Indonesia; 7 World Health Organization, Country Office for Thailand, Nonthaburi, Thailand; 8 Department of Clinical Tropical Medicine, Faculty of Tropical Medicine, Mahidol University, Ratchathewi, Bangkok, 10400 Thailand; 9 Mahidol-Oxford Tropical Medicine Research Unit, Faculty of Tropical Medicine, Mahidol University, Bangkok, Thailand; 10 Centre for Tropical Medicine and Global Health, Nuffield Department of Medicine, University of Oxford, Oxford, UK; 11 Department of Parasitology, Faculty of Medicine, The University of Hasanuddin, Makassar, Indonesia

**Keywords:** *Plasmodium falciparum*, Resistance, *Pfk13*, *Pfmdr1*, *Pfcrt*, *Pfpm2/3*

## Abstract

**Background:**

Dihydroartemisinin–piperaquine has been Indonesia’s first-line anti-malarial treatment since 2008. Annual therapeutic efficacy studies (TES) done in the last 12 years showed continued high treatment efficacy in uncomplicated *Plasmodium falciparum* malaria. Although these studies did not show evidence for artemisinin resistance, a slight increase in Late Treatment Failure was observed over time. It is highlight to explore the evolution of genetic markers for ACT partner drug resistance since adopting DHA–PPQ.

**Methods:**

Dry blood spots were identified from a mass blood survey of uncomplicated falciparum malaria patients (N = 50) in Sumba from 2010 to 2018. Analysis of genotypic profile (N = 51) and a Therapeutic Efficacy Study (TES) from Papua (N = 142) from 2020 to 2021, 42-day follow-up. PCR correction using *msp1*, *msp2*, and *glurp* was used to distinguish recrudescence and reinfection. Parasite DNA from DBSs was used for genotyping molecular markers for antimalaria drug resistance, including in *Pfk13*, *pfcrt*, and *pfmdr1*, as well as gene copy number variation in *pfpm2/3* and *pfmdr1*.

**Results:**

The study revealed the absence of SNPs associated with ART resistance and several novel SNPs such as L396F, I526V, M579I and N537S (4.25%). In Sumba, the mutant haplotype SDD of *pfmdr1* was found in one-third of the isolates, while only 8.9% in Papua. None of the *pfcrt* mutations linked to piperaquine resistance were observed, but 71% of isolates had *pfcrt* I356L. Amplification of the *pfpm2/3* genes was in Sumba (17.02%) and Papua (13.7%), while *pfmdr1* copy number prevalence was low (3.8%) in both areas. For the TES study, ten recurrences of infection were observed on days 28, 35, and 42. Late parasitological failure (LPF) was observed in 10/117 (8.5%) subjects by microscopy. PCR correction revealed that all nine cases were re-infections and one was confirmed as recrudescence.

**Conclusion:**

This study revealed that DHA–PPQ is still highly effective against *P. falciparum*. The genetic architecture of the parasite *P. falciparum* isolates during 2010–2021 revealed single copy of *Pfpm2* and *pfmdr1* were highly prevalent. The slight increase in DHA–PPQ LTF alerts researchers to start testing other ACTs as alternatives to DHA–PPQ for baseline data in order to get a chance of achieving malaria elimination wants by 2030.

**Graphical Abstract:**

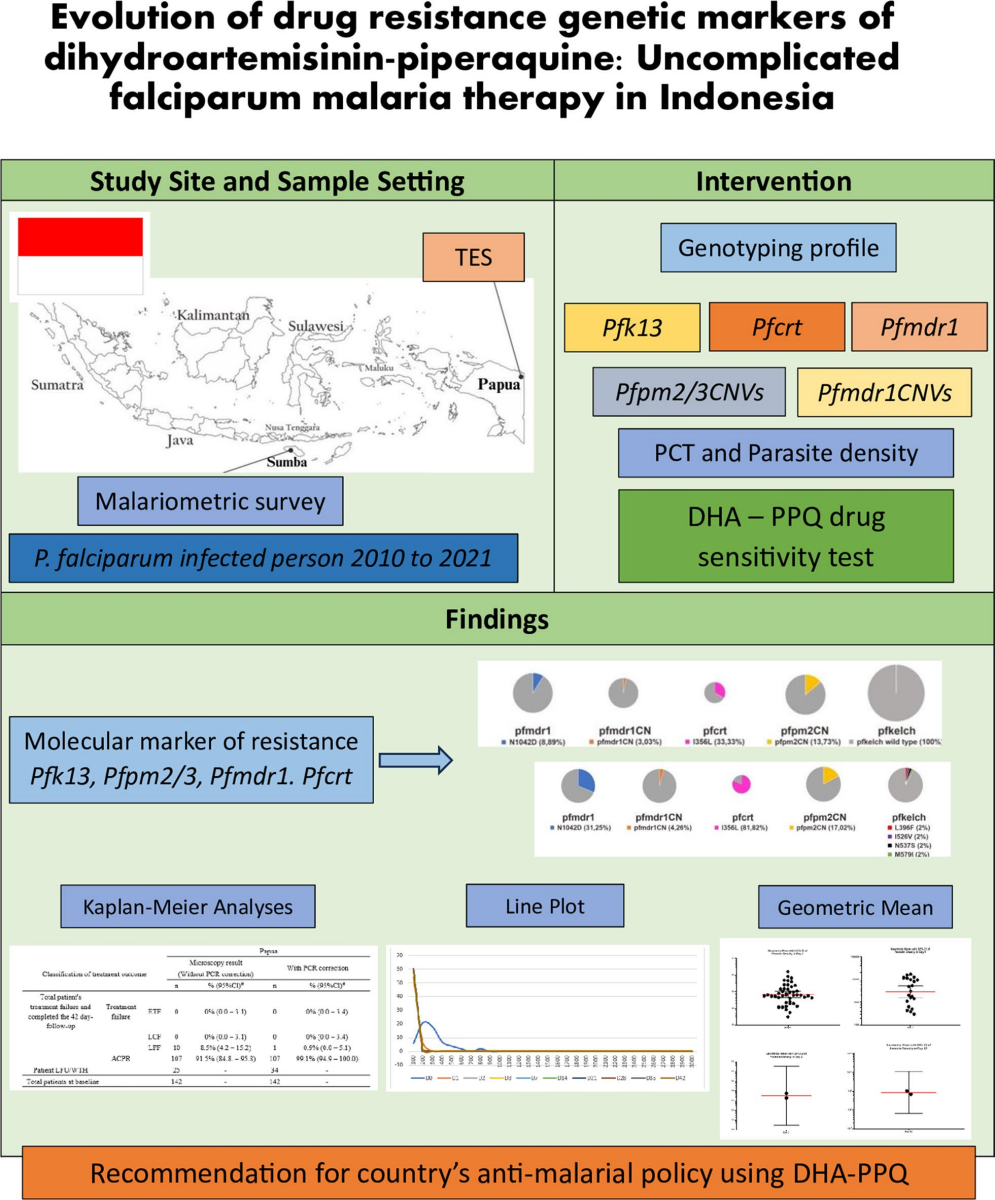

**Supplementary Information:**

The online version contains supplementary material available at 10.1186/s12936-023-04658-4.

## Background

In Indonesia, increasing treatment failure rates in the treatment of uncomplicated falciparum malaria with chloroquine (CQ) and sulfadoxine–pyrimethamine (SP) has prompted a change to artemisinin-based combination therapy (ACT) as first-line anti-malarial treatment since 2004 [1]. ACT combines a potent but short-acting artemisinin (ART) component with a less potent but long-acting partner drug [2, 3]. Artesunate–amodiaquine (AS–AQ) was introduced first, but reports on poor tolerability and increasing treatment failure rates led to changing it to DHA–PPQ in 2008 [4–7].

**Fig. 1:**
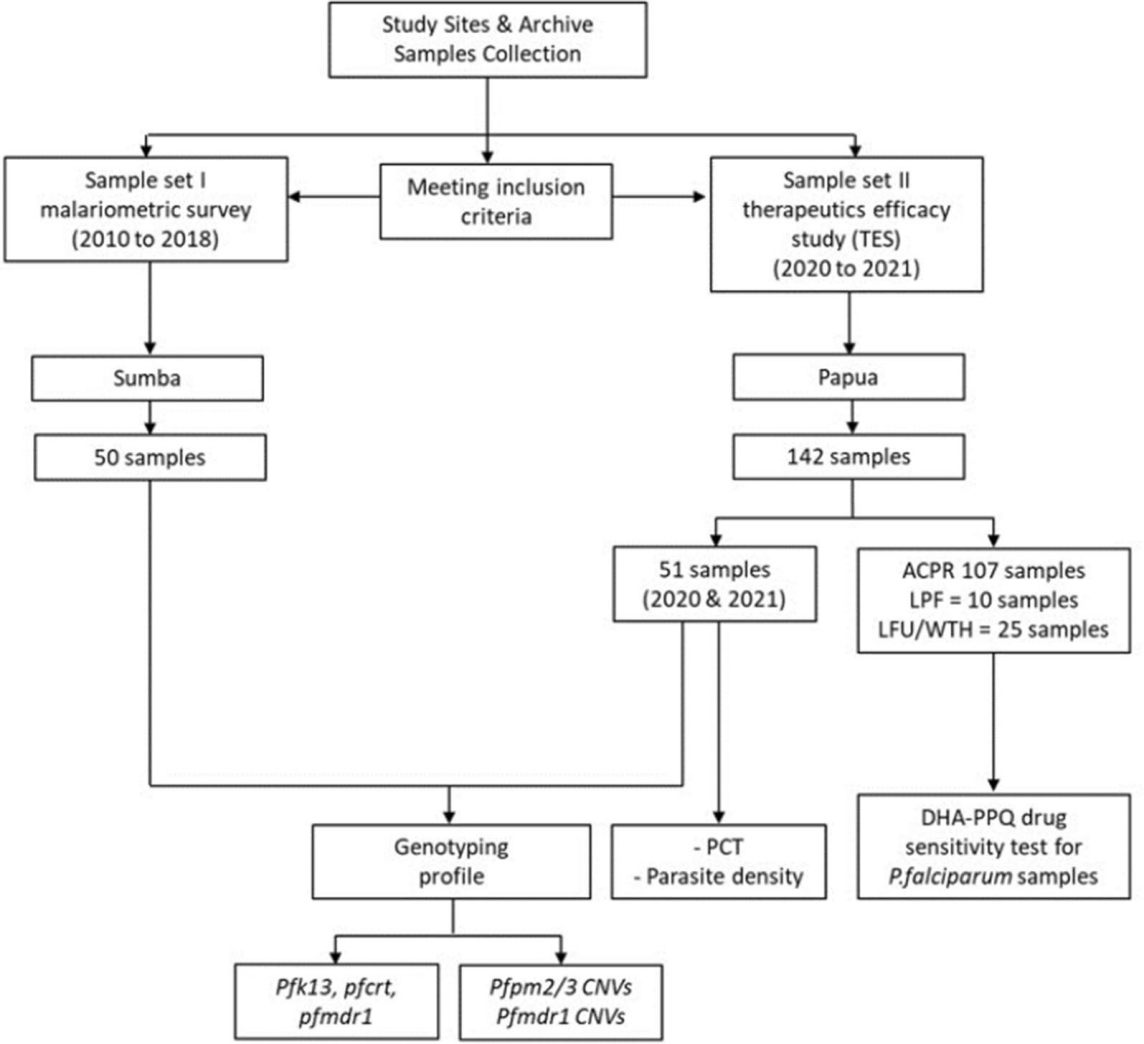
Flowchart for samples set study

Since 2010, this well-tolerated and effective regimen has also become the first-line treatment of other human malaria species, including *Plasmodium vivax*, due to increasing CQ resistance in this species [8]. Studies conducted between 1995 and 2002 in North Sumatra, West Kalimantan, North Sulawesi, West Nusa Tenggara, East Nusa Tenggara, and Papua have consistently demonstrated treatment failure for CQ in *P. vivax* infections. Since then, CQ has not been an effective treatment for acute vivax malaria [9–15]. Several studies have [4, 9, 16] documented excellent effectivity and tolerability for the DHA–PPQ treatment of uncomplicated malaria in Indonesia, including in Papua, Indonesia [17]. Artemisinin resistance in *Plasmodium falciparum* has not been established in Indonesia until now (Fig. 1).

Therapeutic efficacy studies (TES) performed between 2011 and 2018 in South Sumatra, Central Kalimantan, West Kalimantan, North Sulawesi, Central Sulawesi, North Maluku, and East Nusa Tenggara have shown consistently high 42-day cure rates with DHA–PPQ for the treatment of uncomplicated falciparum malaria. No evidence was found for delayed parasite clearance, the hallmark of ART resistance [17]. From 2017 to 2018, molecular surveillance of K13 in Papua New Guinea samples revealed that all *P. falciparum* isolates carried the wild-type allele of K13 [18, 19]. Some other mutations, such as G453W (20%), V454C (20%), E455K (20%), and T474A (2.6%), were also observed at low frequencies [20]. An increased copy number of the *pfpm2/3* gene has been detected in some Papua isolates surviving the DHA–PPQ [5].

Even though DHA–PPQ are still efficacious in treating malaria patients and there was no evidence of treatment failure, evaluation study for genotypic profile and additional measures, such as parasite clearance time (PCT) and parasite density should be regularly monitored. The interval between the patient’s first dose to the time of the first negative blood slide was called the PCT. According to a study in Papua, from April 2017 to April 2018, recurrent *P. falciparum* parasites were detected in 7 out of 102 cases that completed the 42-day follow-up and were classified as LTF at days 21, 35, and 42. Of the 7 LTF cases, one was re-infected with *P. vivax*, 2 were confirmed as recrudescent infections, and the remaining 4 were re-infections. No delay in parasite clearance or severe adverse reaction was observed in any study participant [5].

The efficacy of ACT depends on the sensitivity of the parasites for both components of the combination. Artemisinin resistance can be monitored by assessing the parasite clearance rate and the presence of SNPs in the *Pfk13* gene, a well-established marker for ART resistance. Resistance to some of the ACT partner drugs can be monitored through molecular surveillance. These include: for PPQ SNPs in the *pfcrt* gene (position 343, 350, 353) and copy number variations (CNVs) of the *P. falciparum Plasmepsin2/3* gene (*pfpm*2/3) and *pfmdr1* gene. In addition for ART SNPs in the *pfcrt* gene (356).

The present study aims to assess the evolution of genetic markers for anti-malarial drug resistance following the adoption of DHA–PPQ as the first-line anti-malarial drug in 2010 for any uncomplicated malaria cases, the temporal dynamics trends of the evolution of *pfk13*, *pfcrt*, *pfmdr1* genes also a copy number of *pfpm2/3* and *pfmdr1* from Sumba and Papua and observation of PCT including parasite density. This risk arises as more parasites may develop resistant to PPQ.

## Methods

### Study sites and sample collection

**Fig. 2:**
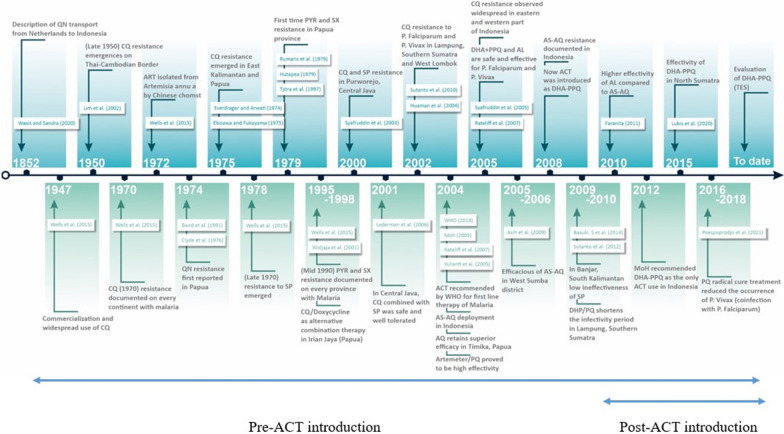
Time points history highlights of antimalaria drug resistance in Indonesia along with Southeast Asia. Cited are studies by Wasis and Sandra [65], MoH [66], Hutapea [67], Tjitra et al. [52], Wells [53], Lim et al. [54], Ebisawa and Fukuyama [68], Rumans et al. [69], WHO [51], Cylde et al. [70], Baird et al. [71], Poespoprodjo et al. [72], Yuliani et al. [73], Ratcliff et al. [4], Lederman et al. [74], Sutanto et al. [75], Syafruddin et al. [48], Syafruddin et al. [33], Basuki et al. [76]

*Plasmodium falciparum* parasite DNA from filter paper blood spots was obtained from two different sample set studies as described in Fig. 2. The selected area for sample set studies was based on the fact that both sites had high annual parasite incidence (API). The total API in Jayapura and Keerom, Papua, from 2019 until 2021 was 95.43 and 383.01, 92.42 and 360.38, 73.08 and 254.93 cases per 1000 population, respectively. In West Sumba and Southwest Sumba, East Nusa Tenggara API in 2019–2021 showed 33.22 and 11.95, 33.09 and 24.21, 16.79 and 10.97 cases, respectively [21].

### Sample set 1 malariometric surveys

Fifty blood blots on filter paper (3 MM; Whatman, Hillsboro, OR, USA) containing approximately 25–50 μL blood equivalent were collected from Sumba during 2010–2018 through active case detection. Sample selection was based on the availability of the existing archived sample.

### Sample set 2 therapeutics efficacy study (TES)

A therapeutic efficacy study was conducted in Papua Province from 2020 to 2021 by the Eijkman Institute for Molecular Biology, National Research and Innovation Agency, Cibinong, Indonesia, serves as part of DBS’s analysis for the current study. TES procedures followed World Health Organization (WHO) criteria. Briefly, smears and blots on filter paper (Whatman International Ltd., Maidstone, UK) were collected from finger pricks on days before enrolment, then on days 1, 2, 3, 7, 14, 21, 28, 35 and 42. The blood-spotted filter papers were allowed to dry completely, transferred into individual plastic bags, labelled, and stored at room temperature in a silica gel desiccator until further processing. 2749 subjects were screened through passive and active case detection, and 42% (1156/2749) were positive for malaria. One hundred forty-two who met the inclusion criteria samples were obtained from 768, a pool of *P. falciparum* malaria-infected individuals. The respondents were aged between 1 and 65 years, weighed more than 5 kg, and had a fever or history of fever in the preceding 24 h, with slide-confirmed malaria with a parasitemia of ≥ 500/µL asexual parasites for *P. falciparum.* Meanwhile, they were excluded with the following exclusion criteria: pregnant, had a history of allergy to the study drugs or studied drug’s derivative, had previously completed treatment with an anti-malarial drug in the preceding 2 weeks, or had a medical history of untreated hypertension or chronic heart, kidney, or liver disease [5]. All study participants were given a supervised treatment of DHA–PPQ from a primary health center containing 40 mg DHA and 320 mg PPQ per tablet and were administered once a day for three days, as a weight per dose regimen of 2.25 and 18 mg/kg of DHA–PPQ [26]. Treatment outcomes and a new infection were classified according to the WHO criteria [16, 17, 22].

In this study, fifty-one archived samples, consisting of 41 successful treatments and 10 treatment failure, were selected from one hundred forty-two subjects to analyze the genotypic profile of molecular markers associated with DHA–PPQ resistance treatment. As part of the Papua TES study, 142 subjects were evaluated for clinical and parasitological efficacy of DHA–PPQ during days 0, 1, 2, 3, 7, 14.21, 28, 35, 42 [22].

### Genomic DNA preparation

DNA from all samples (including sample set 2, on the day of enrolment and day of recurrence) were extracted from the DBS samples using a Chelex-100 ion exchanger (Biorad Laboratories, Hercules, CA, USA) [22]. The genomic DNA obtained was purified following the Qiagen procedures.

### Evaluation of mutations in *Pfk13*, *Pfcrt* and *Pfmdr1*

Polymorphisms in the *Pfk13* gene were investigated using nested PCR amplification covering the gene’s propeller region [23], followed by sequencing with an ABI sequencer (Macrogen Inc, South Korea). The sequencing results were then aligned against the reference strain 3D7’s *Pfk13* gene (PF13 0238) (NCBI reference sequence no. XM 001350122.1). The analysis was carried out using the BioEdit software (Abbott, CA, USA).

PCR: *pfcrt* was amplified from the DNA template to assess *pfcrt* mutations linked to PPQ resistance identified in a previous study [24, 25]. These were codons 343, 350, 353 and 356. On a 2% agarose gel, PCR product were visualized.

*Pfmdr1* was amplified from the DNA template using nested PCR to assess *pfmdr1* mutations, including the following SNPs: 1034, 1042 and 1246. The PCR amplicon was analysed on a 3% agarose gel under ultraviolet illumination. All PCR products were sent for DNA sequencing at 1^st^ Base Inc. in Singapore for quality control [26, 27]. Details of amplification primers sequences and PCR product results are available in Additional file 1: Table S1, Fig. S1.

### Assessment of *PfPlasmepsin2/3* and *pfmdr1* gene amplification

Relative quantitative real-time PCR (TaqMan real-time PCR) on an Applied Biosystems 7500 quantified *pfpm 2/3* and *pfmdr1* copy numbers (Roche Molecular Systems, Inc., USA). Previously disclosed primers and probes [28, 29], a BioRad CFX 96 thermocycler was used to amplify 20 μL in triplicate. Copy number estimates were 2−∆∆CT, where CT is the difference between the unknown sample’s threshold cycle (CT) and the reference sample’s CT. Runs are not interpretable of Ct values > 33 for *pfpm2/3* or *pftub* or sample with a copy number estimate of < 0.5; reactions were repeated. As in previous studies, the main analysis used a cut-off copy number estimate of 1.5 to distinguish single-copy from multiple-copy *pfpm2/3* and *pfmdr1* gene carriage [28, 29].

### Method for distinguishing between recrudescence and re-infection

The *Plasmodium* speciation and genotype of *P. falciparum* were determined using PCR. Genotypic analyses of the parasites at day 0 and the day of recurrence were conducted using the three markers recommended by the WHO: merozoite surface protein 1 (MSP1), MSP2, and glutamate-rich protein (GLURP) genes [26, 27]. Cases were categorized as re-infections as the genotypes of the parasites found on the day of recurrence differed from those found on day 0 (pre-treatment). The identical genotypes for the three markers could be either recrudescent [25, 28].

### Analysis

Analysis was performed using Microsoft Office Excel basic functions and open-source software, RStudio version 2022.07.2+576, based on R version 4.2.2 [30, 31]. Significant differences in SNPs prevalence proportions each year during the study period were analyzed using the Fischer exact test for categorical variables or the Mann–Whitney U test for nonparametric comparisons. This study used the Excel Kaplan–Meier analysis template provided by the WHO. The results are expressed as success and failure cumulative incidence, with 95% CI.

## Results

Demographic characteristics for TES samples as shown in Table 1. Dry blood spots from a mass blood survey of uncomplicated falciparum malaria patients (N = 50) in Sumba from 2010 to 2018 and fifty-one from TES from 2020 to 2021 in Papua to analyze the genotypic profile of molecular markers associated with DHA–PPQ resistance treatment. As part of the Papua TES study, one hundred forty-two subjects were evaluated for clinical and parasitological efficacy of DHA–PPQ during a 42-day follow-up (Table 2). No early treatment failure (ETF) was observed in Papua. However, ten patients out of 117 (8.5%) had a recurrent infection on days 28, 35 and 42 as late treatment failure (LTF) (Table 3).

**Table 1:** Demographic characteristics from Papua TES samples

Variable	Overall cases
Number of persons enrolled	142
Age group (years)	
Mean (SD)	16.5 (12.4)
Range (y.o):	2–53
Adults	7
5 to 15	34
Under 5	6
Gender	
Male [n (%)]	72 (50.7%)
Female [n (%)]	70 (49.3%)
Body temperature [°C, mean (SD)]	
Mean (SD)	37.9 (1.1)
Range	36–39.8

**Table 2:** Treatment outcome from Papua TES during the 42 day of follow-up

Classification of treatment outcome	Papua			
	Microscopy result (without PCR correction)		With PCR correction	
	n	% (95% CI)^a^	n	% (95% CI)^a^
Total patient’s treatment failure and completed the 42 day-follow-up				
Treatment failure				
ETF	0	0% (0.0–3.1)	0	0% (0.0–3.4)
LCF	0	0% (0.0–3.1)	0	0% (0.0–3.4)
LPF	10	8.5% (4.2–15.2)	1	0.9% (0.0–5.1)
ACPR	107	91.5% (84.8–95.8)	107	99.1% (94.9–100.0)
Patient LFU/WTH	25	–	34	–
Total patients at baseline	142	–	142	–

*ETF* early treatment failure, *LCF* late clinical failure, *LPF* late parasitological failure, *LFU* lost to follow up, *WTH* withdrawn

^a^Kaplan–Meier analysis

**Table 3:** Genotyping results of the parasites at day 0 and day of recurrence in *P. falciparum* Papua TES

Isolate code	D0 strain MSP1^a^/MSP2^b^/GLURP^c^	DR strain MSP1^a^/MSP2/^b^GLURP^c^	Day of recurrence	Recurrent/reinfection
PAF 204	K1/FC27/Code1	K1/FC27/Code1	D42	Recrudescent
PAF 205	K1/FC27/Code2	K1-RO33/FC27/Code3	D42	Reinfection
PAF 213	K1/FC27/Code1	K1/3D7/Code1	D42	Reinfection
PAF 221	K1/FC27/Code2	K1-RO33/FC27/Code3	D28	Reinfection
PAF 222	K1/FC27/Code3	MAD20/3D7/Code3	D42	Reinfection
PAF 266	K1/FC27/Code1	K1/3D7/Code1	D28	Reinfection
PAF 274	K1/MAD20/FC27Code2	RO33/3D7/Code3	D28	Reinfection
PAF 307	K1/FC27/Code3	MAD20/3D7/Code3	D35	Reinfection
PAF 335	K1/FC27/Code1	K1/3D7/Code1	D28	Reinfection
PAF 358	K1/MAD20/FC27Code2	RO33/3D7/Code3	D42	Reinfection

^a^MSP1 amplicon: K1 = 150–300 base pairs (bp); MAD20 = 150–400 bp; and RO33 = 120–230 bp

^b^MSP2 amplicon: FC27 = 250–700 bp; 3D7 = 280–780 bp

^c^GLURP amplicon: Code1 = 501–600 bp; Code2 = 601–700 bp; and Code3 = 701–800 bp

### Single nucleotide polymorphisms in the *Pfk13* gene

**Fig. 3:**
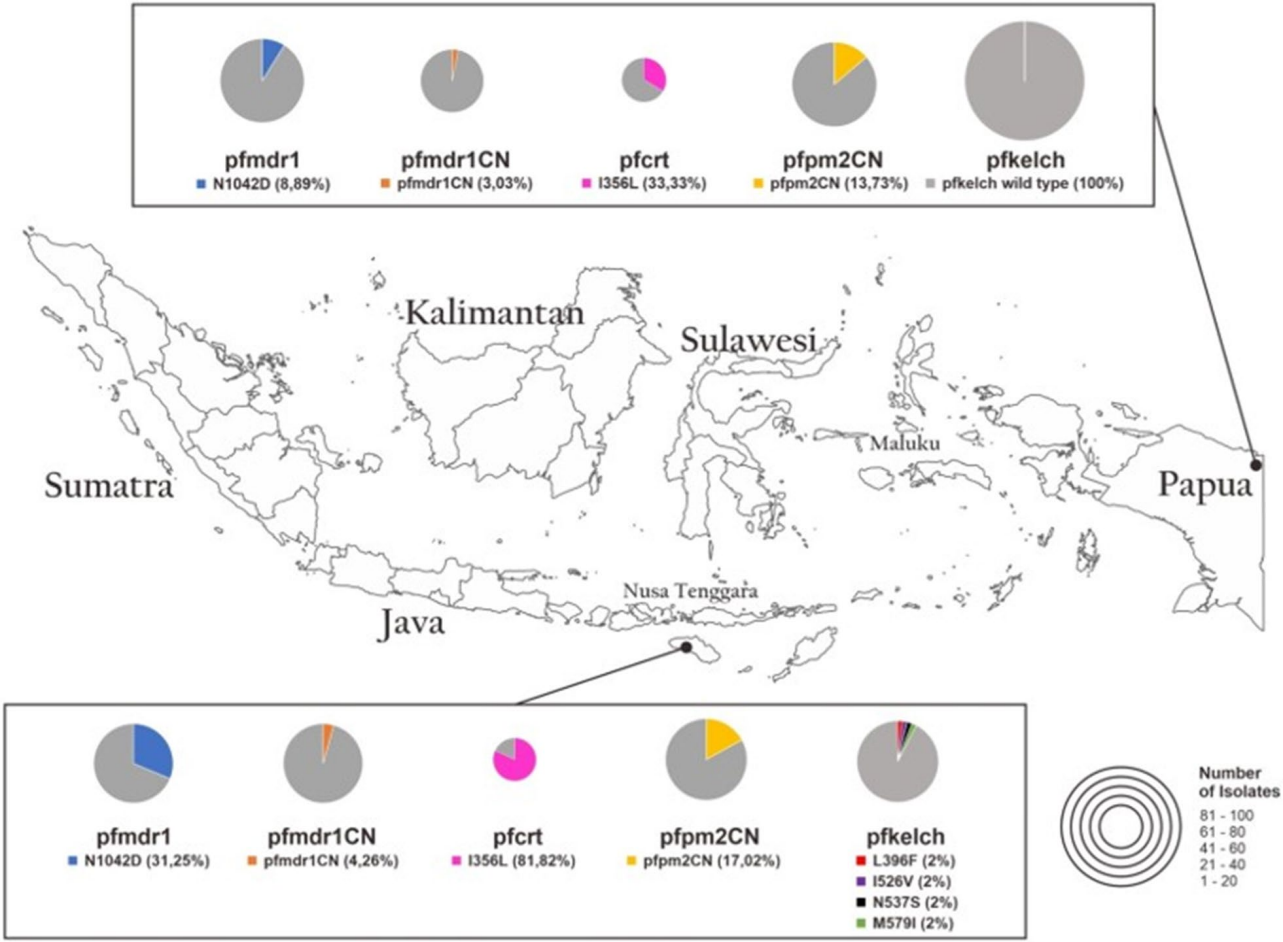
Map of Indonesia indicating molecular marker of resistance and sampling locations of the *P. falciparum* field isolates during observation study. Map source from Natural Earth (https://www.naturalearthdata.com) and modified according to data from the references

Of the 101 DNA specimens analysed, 94 gave full amplicons. None of the 20 SNPs previously reported to be associated with ART resistance were found. As shown in Fig. 3, the overall prevalence of the non-synonymous new mutant allele in BTB/POZ and the propeller domain was found in Sumba with a percentage of 4/94 (4.25%, 95% CI 0.94–1; see Additional file 1: Fig. S2, Table S2; Table 4) at positions L396**F**, I526**V**, N537**S** and M579**I**.

**Table 4:** Summary of *PfK13*, *pfcrt*, *pfmdr1* SNP frequencies, *pfpm2/3* and *pfmdr1* CNVs in Sumba and Papua

Gene	Haplotype	(All) total		Total Sumba		Sumba														Total Papua		Papua			
						Year 2010		2011		2012		2015		2016		2017		2018				Year 2020		2021	
		Number	%^a^	Number	%^a^	Number	%^a^	Number	%^a^	Number	%^a^	Number	%^a^	Number	%^a^	Number	%^a^	Number	%^a^	Number	%^a^	Number	%^a^	Number	%^a^
*PfK13* Total n = 101	L396**F**	1	1	1	20	0	0	0	0	1	20	0	0	0	0	0	0	0	0	0	0	0	0	0	0
	I526**V**	1	1	1	16.7	0	0	0	0	0	0	0	0	0	0	0	0	1	16.7	0	0	0	0	0	0
	N537**S**	1	1	1	20	0	0	0	0	1	20	0	0	0	0	0	0	0	0	0	0	0	0	0	0
	M579**I**	1	1	1	20	0	0	0	0	1	20	0	0	0	0	0	0	0	0	0	0	0	0	0	0
	WT	97	96	46	92	20	100	5	100	2	40	5	100	4	100	5	100	6	100	51	100	27	100	24	100
*pfcrt* Total n: 31	I356**L**	22	71	18	95	3	15	3	60	4	80	3	60	2	50	3	60	0	0	4	33.3	2	50	2	25
	WT	9	29	1	5.3	17	85	2	40	1	20	2	40	2	50	2	40	0	0	8	66.7	2	50	6	75
*pfmdr1* Total n = 93 Positions 1034, 1042, 1246	SDD	19	20.4	15	31.3	3	15.8	4	80	2	40	3	75	1	33.3	2	40	0	0	4	8.9	3	10.3	1	6.3
	SND	74	79.6	33	68.8	16	84.2	1	20	3	60	1	25	2	66.7	3	60	5	100	41	91.1	26	89.7	15	93.8
*pfpm2/3 CNV* Total n = 98	Single copy	83	84.7	39	83.0	0	0	0	0	0	0	0	0	3	75	2	40	2	33.3	44	86.3	24	77.4	20	100
	Multiple copies	15	15.3	8	17.0	0	0	0	0	0	0	0	0	1	25	3	60	4	66.7	7	13.7	7	22.6	0	0
*pfmdr1 CNV* Total n = 80	Single copy	76	95.0	45	95.7	0	0	4	80	4	80	0	0	0	0	0	0	0	0	31	94	18	94.7	14	100
	Multiple copies	3	3.8	2	4.3	0	0	1	20	1	20	0	0	0	0	0	0	0	0	1	3	1	5.3	0	0

^a^Frequency data are presented as the percentage of sequences that carried a mutation out of the total number successfully sequence

### *Pfplasmepsin 2/3 *and *Pfmdr1* gene amplification

*Pfplasmepsine 2/3* copy numbers were measured successfully in 98 samples. Since 2010, the National Malaria Control Programme, the Ministry of Health, Republic of Indonesia, has recommended DHA–PPQ as the first-line drug for uncomplicated malaria. After the deployment of DHA–PPQ, the prevalence of parasites with *pfpm2/3* amplified slowly during 2016–2018 in Sumba (Table 4). Amplification of the *pfpm2/3* genes in Sumba and Papua was found at 8/47 (17.02%) and 7/51 (13.7%), respectively (Table 4). Ten recurrent isolates from Papua showed no *pfpm2/3* amplification (Table 5).

**Table 5:** Summary of ten recurrent samples *pfK13*, *pfmdr1* SNPs mutation, *pfpm2/3* and *pfmdr1* CNVs in Papua

No.	ID sample	*PfK13*	*pfmdr1* SDD	*pfpm2/3 CNVs*	*pfmdr1 CNVs*
1	PAF 204	WT	SND	Single copy	–
2	PAF 205	WT	SND	Single copy	–
3	PAF 213	WT	SND	Single copy	–
4	PAF 221	WT	SND	Single copy	Single copy
5	PAF 222	WT	SND	Single copy	Single copy
6	PAF 266	WT	–	Single copy	Single copy
7	PAF 274	WT	–	Single copy	Single copy
8	PAF 307	WT	–	Single copy	–
9	PAF 335	WT	–	Single copy	–
10	PAF 358	WT	SDD	Single copy	–

Eighty samples were successfully measured for *pfmdr1* copy numbers, of which 3/80 (3.8%) were multiple copies. In Sumba and Papua, parasites with *pfmdr1* amplification tend to disappeared after AS–AQ was replaced with DHA–PPQ in 2010 (Table 4). This study did not observe the  concomitant amplification of both *pfpm2/3 and pfmdr1* (Table 5).

### Single nucleotide polymorphisms in the *pfcrt* gene

Only 31 of the 101 specimens were successfully PCR amplified for the *pfcrt* gene due to a lack of parasite DNA. None of the *pfcrt* mutations linked to piperaquine resistance were observed. Although the number of samples was small, approximately 71% (22/31) of all isolates from both study areas had *pfcrt* I356L, as shown in Table 4 and Additional file 1: Table S2.

### Single nucleotide polymorphisms in the *pfmdr1* gene

PCR amplicons of *pfmdr1* were amplified from 90 samples (codons 1034, 1042, and 1246) obtained. Table 4 and Additional file 1: Table S2 show that approximately 79.6% (74/93) of isolates had the wild-type SND haplotype. In Sumba, the mutant haplotype SDD was found in one-third of the isolates (15/48; 31.3%); in Papua, the haplotype was found in 8.9% (4/45). In Papua, during 2 years of observation, prevalence decreased from 10.3% (3 of 29) to 6.3% (1 of 16) (Table 4). As both alleles (1034 and 1246) were observed at multiple locations in Indonesia across the region [32, 33], similar observations in *pfmdr1* (1034**C** and 1246**Y**) were not found in any of the isolates examined.

### Clinical and parasitological characteristics of Papuan TES

Analysis of one hundred forty-two *P. falciparum*-infected subjects (Table 1) 117 cases completed the 42 day-follow-up, and 25 cases were either lost to follow-up (LFU) or withdrawn (WTH). The classification of the treatment outcomes by PCR correction is presented in Table 3. At day 42, ACPR was noted in 91.5% (95% CI 84.8–95.8). Of the 10 LTF cases, nine were re-infections and one was confirmed as recrudescent (Table 3). Therefore, the PCR-corrected DHA–PPQ efficacy for falciparum was 99.1% (95% CI 94.9–100.0). No delay in parasite clearance at day 3 was observed in any isolates.

The PCT ranged from 1.5 to 35.7 days, with a median of 1 day (interquartile range [IQR], 1 to 2 days) and 38.5 days (IQR, 28 to 42 days) in the ACPR and recurrence groups, respectively. The dynamics of parasite density based on frequencies depict a decreasing trend during observations (Additional file 1: Fig. S3), although only one sample on the last day of observations had high parasitaemia. The imputed corrected geometric mean parasite densities (/μL) for detected infections were 707,326 parasites/mL (95% CI 469,080–1,066,577 parasites/mL) with standard deviation s = 2,891,848 (IQR: 299,600–1,525,600) in D0; 29,078 parasites/mL (95% CI 15,665–53,975 parasites/mL) with standard deviation s = 55,888 (IQR: 8800–106,800) in D1; 3326 parasites/mL (95% CI 3095–3,573,239 parasites/mL) with standard deviation s = 2715 (IQR, 1920–5760) in D2; 47,614 parasites/mL (95% CI 0.12–18 × 10^10^ parasites/mL) with standard deviation s = 80,554 (IQR, 17,280–131,200) in D42 (Additional file 1: Fig. S4).

## Discussion

In Indonesia, AS–AQ was introduced in 2004 and poor tolerability was reported. Increasing treatment failure rates of AS–AQ led to drug policy change to DHA–PPQ in 2008. DHA–PPQ resistance has emerged in South-East Asia, posing a significant threat to malaria control and elimination efforts [34–36]. DHA–PPQ is well tolerated, with a faster ART derivative clearing parasites and the active compound PPQ removing the remaining parasites more slowly [37]. TES conducted in several parts of Indonesia [17, 19] revealed that DHA–PPQ is still highly effective, evidenced by the absence of delayed parasite clearance and low cases of parasite recurrence after 42-day observations.

Analysis of the *pfk13* gene of *P. falciparum* isolates collected from 2010 to 2021 revealed the absence of mutations associated with ART resistance. However, several polymorphisms, such as L396F, I526V, N537S, and M579I, were found. Although not associated with ART resistance, these SNPs are newly found and have never been described in any endemic area [16, 18–20, 23, 24, 34–50]. It is still important to describe further the roles of the four SNPs. The average PCT result of 1.5 days and no delay in parasite clearance at day 3 was observed in any isolates. The TES results from Papua do not meet the WHO criteria for suspected ART resistance [51].

Resistance to PPQ has been associated with the increasing copy number of the *pfpm2* gene [28, 52, 53] and more recently with mutant alleles of *pfcrt* [54–58]. This research result on 10 recurrent infection cases revealed no association of PPQ resistance with *pfpm2/3* copy number (Table 5). Prior TES analysis in the southern region of Papua also did not identify any *P. falciparum* isolates carrying multiple copies of the *pfpm2/3 gene* [18]. Other studies proposed *pfcrt* mutations associated with PPQ resistance, namely 343, 350, or 353 [24, 25]. Those mutations were not observed in this study. After the deployment of DHA–PPQ, the prevalence of parasites with multiple copies of *pfpm2/3* slowly increased from 2016 to 2018 and seemed to be relatively prevalent in no recurrent isolates. All this happened probably due to the selective exposure of DHA–PPQ over more than 10 years of adoption in Indonesia. The limited number of samples used in this study could also be a determining factor, so it will need further investigation to confirm. Although there was no evidence of an increased copy number of *pfpm2/3* in recrudescence cases, the PPQ treatment still failed to eradicate the parasite from the blood and prevent reinfection during the follow-up period.

Surveillance in areas relying on DHA–PPQ as first-line anti-malarial treatment indicated that *pfpm2/3* amplification was not the sole factor rendering PPQ resistance. The genetic background of circulating field isolates appeared to play a role in drug susceptibility [36, 37]. It is also supported by Fidock et al*.* [42] and Iwanaga et al*.* [59], specifically revealing that a drug-resistant strain’s successful production is directly generated in a drug-sensitive strain via in vitro study or genome-wide functional screening of drug resistance. These transformations were influenced by geographical origin (South East Asia, Africa and South America) and genetic background (haplotype allele or genotype of all other related genes), supporting the population survey results that the mutated *pfcrt* was possibly sufficient to confer resistance [60]. It was also suggested that the initial selection of *pfpm2/3* and *pfmdr1* CNVs, although a PPQ-resistant phenotype does not emerge, it developed a genetic background for novel *pfcrt* mutations [61].

*Pfcrt* is a 13-exon gene with several point mutations 74, 75, 76, 220, 271, 326, 356, 371 that connect with CQR [42] and 93, 97, 145, 218, 343, 350, 353 exclusively associated with PPQ resistance [36, 60, 62] located on chromosome 7 spanning from 36 kb segment. The overlapped region was correlated to the *pfcrt* gene and its regulatory elements such as the promoter and 3′ untranslated region (3′ UTR) responsible for regulating and activating the coding region [59]. The mutations in *pfcrt* might interfere with transporting the natural substrates out of the digestive vacuole, resulting in increased osmotic pressure. This phenotype was also observed in Dd2 parasites expressing the *pfcrt* mutations F145I, M343L, and G353V [60]. Not all novel *pfcrt* mutations exhibit a swollen DV phenotype, depending on the location of the mutated amino acids [63].

Intriguingly, *pfcrt* I356T/L mutation also increased ART IC50 values and resistance [38, 39], emphasizing the recent correlation of the I356T mutation in Southeast Asia with ART-resistant parasite populations in the *pfk13* mutation.[40, 41]. Several *pfcrt* haplotype lines from many geographic regions serve as genetic backgrounds, with 356 alleles as one of them was associated with the development of ART resistance in *P*. *falciparum* parasites [38–40, 42]. The presence of moderate frequencies of *pfcrt* I356L 22/31 (71%) in the study site was possibly associated with long-time drug-selected pressure from DHA–PPQ treatment used and the availability of access to CQ in the private health sectors (non-malaria purposes) that could facilitate the evolution of ART resistance *pfcrt* alleles [16, 39].

*Pfmdr1* was also proposed as a modulator for PPQ resistance. [28, 34] Resistance to PPQ was also associated with the amplification of *pfmdr1*. Another in-vitro study reported a correlation between a single copy number of *pfmdr1* and *P. falciparum* isolates resistance to PPQ [35, 36, 43]. *Plasmodium falciparum* parasites might suffer a fitness disadvantage or reduced transmissibility if *pfmdr1* gene is amplified more frequently [47]. By contrast, the multiple copy number of *pfmdr1* was associated with MQ resistance [29, 37, 44, 64]. This study revealed that *P. falciparum* isolates in Indonesia predominantly posed single copy of *pfmdr1* 95%, suggesting reduced PPQ effectiveness. Previous studies observed a reduced prevalence of multicopy *pfmdr1* since adopting DHA–PPQ [28, 37, 45]. However, the role of *pfmdr1* remained controversial [43, 46].

Besides decreasing CNVs of *pfmdr1*, polymorphism in the *pfmdr1* gene, such as N1042**D** increased by 76.9% before the introduction of PPQ treatment. The result in another eastern part of Indonesia SNP mutation S1034**C** was observed to reach 100% in 2010 [49]. After PPQ was introduced, the *pfmdr1* S1034**C**, N1042**D** and D1246**Y** decreased significantly by 20.4%. There was a notable change in haplotype frequencies between the SND haplotype and mutant SDD haplotype (Table 4).

## Conclusions

This study revealed that DHA–PPQ is still highly effective against *P. falciparum*. The genetic architecture of the parasite *P. falciparum* isolates during 2010–2021 revealed that *pfmdr1* and *pfpm2/3* single copy number were highly prevalent. The slight increase in DHA–PPQ LTF alerts researchers to the need to test alternative ACTs for baseline information in the event that DHA–PPQ will need to be replaced.

### Supplementary Information

**Additional file 1: Table S1.** Primer sequences in this study. **Figure S1.** PCR product result for (A) K13^1^ propeller domain (B) *pfcrt* exon 10^2^ (C) *pfmdr1*^3^ in electrophorese gel 2%. **Figure S2.** Chromatograms of sequence analysis on mutation position of the *Kelch13* BTB/POZ and propeller domain in Sumba. The arrow shows the mutation position. **Table S2.** Summary of Confidence Interval (CI) 95% with lower and upper CI in each molecular marker associated with drug resistance. **Figure S3.** Line plot showing parasite density based on frequency per day observations in Papua. **Figure S4.** Geometric mean of parasite density during observation period 2020 to 2021 in Papua.

## Data Availability

All data generated or analysed during this study are included in this published article and its Additional files.

## Abbreviations

*P.** falciparum*: *Plasmodium falciparum*
ACT: Artemisinin combination therapy
DHA–PPQ: Dihydroartemisinin–piperaquine
PPQ: Piperaquine
CQ: Chloroquine
SP: Sulfadoxine–pyrimethamine
ART: Artemisinin
LUM: Lumefantrine
AS–AQ: Artesunate–amodiaquine
*PfK13*: *Plasmodium falciparum Kelch 13*
*Pfcrt*: *Plasmodium falciparum chloroquine resistance transporter*
*Pfmdr1*: *Plasmodium falciparum multidrug resistance 1*
*Pfpm2/3*: *Plasmodium falciparum plasmepsine 2/3*
TES: Therapeutic Efficacy Study
LTF: Late treatment failure
ETF: Early treatment failure
LCF: Late clinical failure
LPF: Late parasitological failure
LFU: Lost to follow up
WTH: Withdrawal
SNP: Single nucleotide polymorphism
DBS: Dry blood spots
MBS: Mass blood survey
PCT: Parasite clearance time
ACPR: Adequate clinical and parasitological responses
API: Annual parasite incidence
CT: Threshold cycle
MSP1: Merozoite surface protein 1
MSP2: Merozoite surface protein 2
GLURP: Glutamate-rich protein
IQR: Interquartile range
