# Supplementary material for: Evolution of genetic markers for drug resistance after the introduction of dihydroartemisinin–piperaquine as first-line anti-malarial treatment for uncomplicated falciparum malaria in Indonesia
Source: Malar J. 2023 Aug 9;22:231. doi: 10.1186/s12936-023-04658-4 (PMC10410932; doi:10.1186/s12936-023-04658-4)
Supplement: Supplementary file 1 — Additional file 1: Table S1. Primer sequences in this study. Figure S1. PCR product result for (A) K131 propeller domain (B) pfcrt exon 102 (C) pfmdr13 in electrophorese gel 2%. Figure S2. Chromatograms of sequence analysis on mutation position of the Kelch13 BTB/POZ and propeller domain in Sumba. The arrow shows the mutation position. Table S2. Summary of Confidence Interval (CI) 95% with lower and upper CI in each molecular marker associated with drug resistance. Figure S3. Line plot showing parasite density based on frequency per day observations in Papua. Figure S4. Geometric mean of parasite density during observation period 2020 to 2021 in Papua. [file 12936_2023_4658_MOESM1_ESM.docx]

**Additional file 1**

**Table S1.** Primer sequences in this study

| **Gene** | **Locus** | **Round** | **Primers** | **Sequence (5’ → 3’)** | **Amplicons (bp)** |
| --- | --- | --- | --- | --- | --- |
| *pfcrt*  (2) | 336-366 | 1^st^ | CRT1-1 | GATTATTTTCATTGTCTTCCACA | 304 |
|  |  |  | CRT1-2 | GATCTCTATACCTTCAACATTATTCC |  |
| *pfmdr1*  (3) | 1034, 1042, 1246 | 1^st^ | MDR1-1 | GTGTATTTGCTGTAAGAGCT | 860 |
|  |  |  | MDR1-2 | GACATATTAAATAACATGGGT TC |  |
|  |  |  | MDR2-1 | CAGATGATG AAATGTTTAAAG ATC |  |
|  |  |  | MDR2-2 | TAAATAACATGGGTTCTTGACT |  |
| *pfK13* (5) | 390 - 620 | 1^st^ | K13-1-1 | CGGAGTGACCAAATCTGGGA | 2063 |
|  |  |  | K13-1-2 | GGGAATCTGGTGGTAACAGC |  |
|  |  | 2^nd^ | K13-2-1 | GCCTTGTTGAAAGAAGCAGA | 849 |
|  |  |  | K13-2-2 | GCCAAGCTGCCATTCATTTG |  |


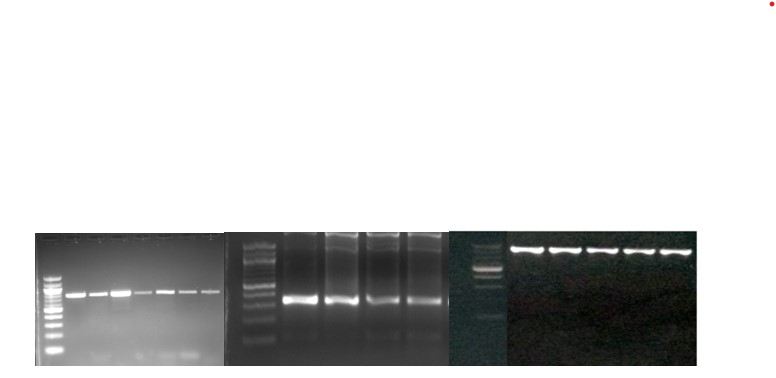


1. (B) (C)

**Figure S1.** PCR product result for (A) K13^1^ propeller domain (B) *pfcrt* exon 10^2^ (C) *pfmdr1*^3^ in electrophorese gel 2%

^1^product size 784 bp

^2^ product size 304 bp

^3^Product size 860 bp


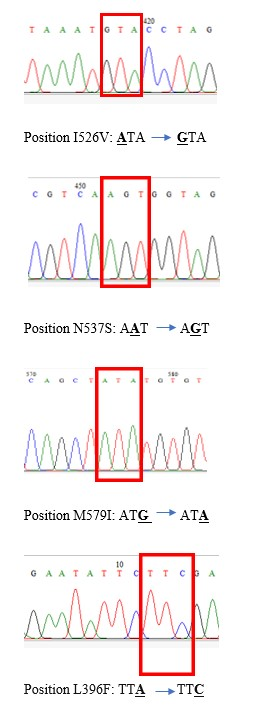


**Figure S2.** Chromatograms of sequence analysis on mutation position of the *Kelch13* BTB/POZ and propeller domain in Sumba. The arrow shows the mutation position.

**Table S2.** Summary of Confidence Interval (CI) 95% with lower and upper CI in each molecular marker associated with drug resistance

| **Molecular Marker** | **Category** | **Estimates** | **Lower CI** | **Upper CI** |
| --- | --- | --- | --- | --- |
| *Kelch13* | Mutation | 0.011 | 0 | 0.6 |
|  | Wild Type | 0.989 | 0.94 | 1 |
| Haplotype *pfmdr1* | SDD | 0.204 | 0.128 | 0.301 |
|  | SND | 0.796 | 0.699 | 0.872 |
| *Pfcrt* I356**L** | 356**L** | 0.71 | 0.52 | 0.858 |
|  | Wild Type | 0.29 | 0.142 | 0.48 |
| *pfpm2* CNVs | Single Copy | 0.847 | 0.760 | 0.912 |
|  | Multiple Copies | 0.153 | 0.088 | 0.240 |
| *pfmdr1* CNVs | Single Copy | 0.962 | 0.894 | 0.992 |
|  | Multiple Copies | 0.038 | 0.008 | 0.106 |


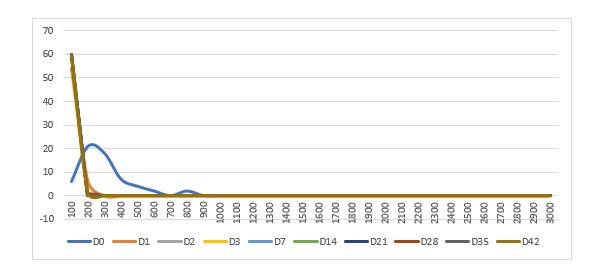


**Figure S3.** Line plot showing parasite density based on frequency per day observations in Papua


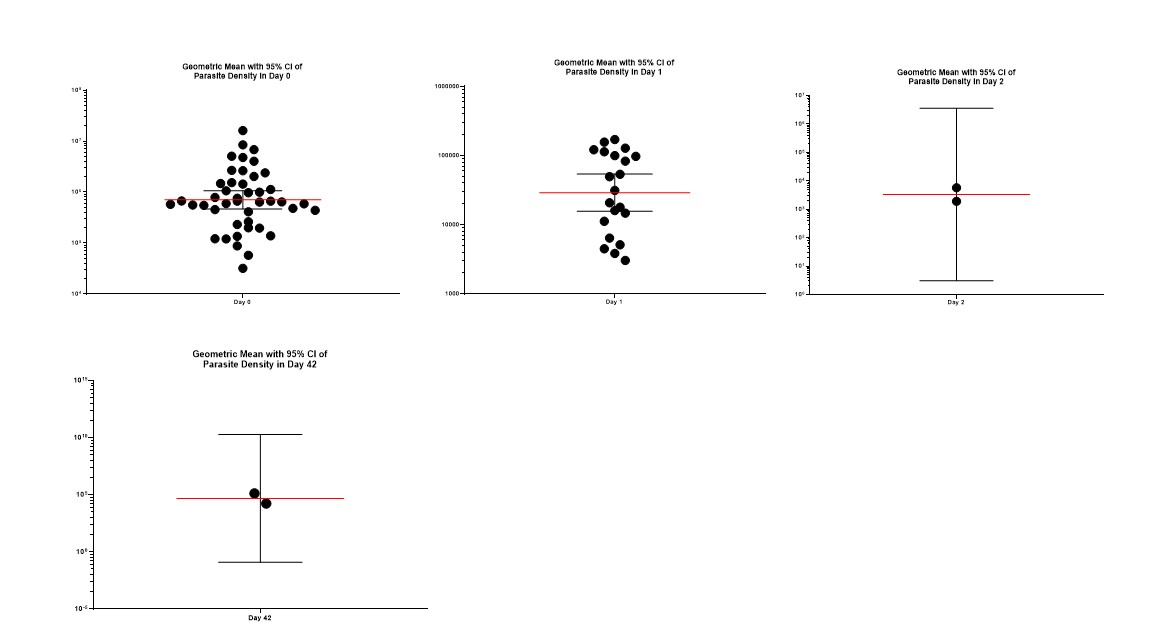


**Figure S4.** Geometric mean of parasite density during observation period 2020 to 2021 in Papua
